# Malignant Growths in the Mouth, Incidental to General Practice

**Published:** 1901-06

**Authors:** George A. Maxfield


### International Dental Journal.

Vol. XXII. June, 1901. No. 6.

### Original Communications.[1]

### MALIGNANT GROWTHS IN THE MOUTH, INCIDENTAL TO GENERAL PRACTICE.[2]

BY GEORGE A. MAXFIELD, D.D.S.

At a meeting of this Institute in April, 1899, Dr. Dawbarn presented a case of sarcoma of the antrum, and commenting on the case Dr. Dawbarn said, "If the dentist who first had the case had recognized the disease and had referred the patient to the proper surgeon, the extensive operation then necessary might have been prevented. As a similar case had come into my hands only a few months previous, a member of your Executive Committee suggested that I make a brief report of a few cases that have come under my observation, thus helping to emphasize the point which Dr. Howe has so often referred to in these meetings,—viz., the necessity of the dentist's ability to recognize malignant growths when first seen, so that the patient may be referred to a competent surgeon in the early stages of the disease.

Although these cases occurring in the mouth and adjacent parts are not common, they are of such rapid growth, generally, that the

[1] The editor and publishers are not responsible for the views of authors of papers published in this department, nor for any claim to novelty, or otherwise, that may be made by them. No papers will be received for this department that have appeared in any other journal published in the country.

[2] Read before The New York Institute of Stomatology, March 5, 1901.

time lost—by ignorance on the part of the dentist in the early stages of the disease—has rendered the life of the patient beyond recovery. Three cases of carcinoma have come into my hands during the past seven years.

CASE I.—March, 1894, Mr. C., farmer, aged seventy, face badly swollen with fistula on under side of jaw directly under the left second molar, which had been discharging for several weeks, came in by advice of his physician to have some teeth extracted, as it had the appearance of an alveolar abscess.

On examination I found the teeth badly affected with pyorrhœa alveolaris and very loose. I extracted two molars and two bicuspids, and a careful examination failed to reveal any connection with the fistula through any of the sockets of the extracted teeth. A probe was then passed into the fistula for over two inches. The patient was referred back to the physician, who sent him to the hospital, where it was pronounced carcinoma. An operation was not thought advisable on account of his advanced age, and he died there some five weeks later.

CASE II.—Mr. W., aged seventy-one, came in the first week of December, 1896, to have a tooth extracted. Examination revealed all the teeth badly affected with pyorrhœa alveolaris, the left inferior second molar quite loose and giving considerable pain, the first molar and both bicuspids missing, and nothing to indicate any other trouble. The second molar was extracted. The second week in January, following, he came in again to have the left inferior cuspid extracted, as this was giving pain. I found it quite loose, and also noticed a small growth—cauliflower-like—in the socket of the molar I had previously extracted. I extracted the cuspid and directed him to call two days later, when I could give him more time. He did not keep the appointment.

Two weeks later, on meeting his niece, I informed her of the serious aspects of the case and the necessity of immediate attention. The day following he came in. I then found the growth in the molar socket had increased in size, projecting above the gum, also a small growth in the socket of the cuspid, the gum near the molar having become ulcerated on the upper surface. The growths bled very easily, and had been staining the pillow every night, and had been giving much pain. I injected the growths with carbolic acid, ninety-five per cent. strength, and removed them, and after thoroughly washing the parts with a weak solution of the acid, I applied a dressing, on a pledget of cotton, of carbolic acid, one part, and glycerin, two parts, and ordered fifteen drops of the same in a glass of water, to be used freely as a mouth-wash. I also prescribed Fellows' hypophosphites. I saw him every two days for a week, when I simply washed the parts with hydrogen dioxide and followed with the same dressing as the first time. The improvement was then so marked that I directed him to continue the treatment at home and to call again in one week.

I next saw him February 18, when I noticed the growth returning in the molar socket. I removed it this time by injecting the tincture of iodine. He then informed me he was going away to visit relatives, as he had suffered no pain since I first removed the growths, nor had there been any hemorrhage. Contrary to my advice, he went away, and I did not see him again until March 12. He was then in excellent spirits and had had no trouble while away. On examination I found the growths had returned, filling both sockets and rolling over the gum, but without ulceration. I then informed him that I was unable to help him, as I felt certain it was a malignant growth. After consulting with his friends he was sent to the Massachusetts General Hospital at Boston, where after a microscopical examination it was pronounced carcinoma, but on account of his age the surgeons did not think it wise to operate. When he returned home, March 15, he assured me he was going to cure the cancer by the use of a decoction made from some roots and herbs, which he called a blood medicine. When I saw him again, six weeks later, May 1, I was surprised to see the marked improvement in his general health, while the condition in the mouth was about the same as when he returned from the hospital. I did not see him again; he died the last of the following November.

Case III.—Mr. B., aged forty-two, was a strong healthy man, who never had had any sickness. While away from home the left superior third molar became somewhat loose, and felt so uncomfortable that he went to a dentist and had it extracted; then, as the troubled feeling continued, he returned to the dentist for treatment. After treating the case for two weeks the dentist extracted the first and second molars adjoining, as he said they were irritating the cheek, told him he had leucoplakia, and continued to treat the case five weeks longer, when the man became so ill that he returned home. The physician called in brought him to me and left him in my charge. This was on December 14, 1898.

On examination I found the cheek somewhat swollen, with inability to open the mouth more than half an inch; the gum on the buccal side much swollen, ulcerated, and hanging down, when the mouth was closed, between the lower teeth and cheek. The alveolus was also exposed and the parts emitting a bad odor. In appearance it resembled cases that have come into my hands of sloughing, as a result of hypodermic injections for extraction, though he assured me that the dentist had not made any injections. At first I thought it might be a case of infection from dental instruments, as the rest of the mouth was in a healthy condition. I washed the parts as thoroughly as possible with hydrogen dioxide and applied the carbolic acid and glycerin. I ordered for home treatment the parts to be sprayed every half-hour through the day with a mixture of equal parts of hydrogen dioxide and Phillips's milk of magnesia, followed by the carbolic mouth-wash. The next day, Thursday, he reported the best night's rest for six weeks. I treated as before, and on Friday there was a marked improvement and absence of all odor. I ordered the home treatment continued, and did not see him again until Monday. I then found the odor returning and pus exuding on the surface. I also noticed on the palatine margin of the gum of the socket of the third molar a small growth, not larger than the head of a pin, cauliflower-like, which at once aroused my suspicion that the trouble was from a malignant growth in the antrum. I ordered the same home treatment continued. When he came in the Wednesday following, there was a slight increase in the size of this growth, and I sent word to his physician.

The following Monday, December 26, we sent him to Dr. Maurice Richardson, of Boston, who confirmed our diagnosis, but said it was too late for an operation, and advised his going to Dr. Coley, of New York, and taking the antitoxin treatment as his only hope. On Thursday he went to Dr. Coley, who, after a microscopical examination, pronounced it carcinoma, but did not give much encouragement regarding the treatment, as the disease had so far advanced. Treatment was begun, however, and at first there appeared to be a marked improvement, but by the end of the third week it was evident the treatment was simply retarding the growth, and he came home the last of January. After this no other treatment was attempted. He died the first day of May, only four months and a half after I first saw him, and only about six months after the first appearance of any trouble with the third molar.

In this case, if the dentist had referred the patient to a surgeon before he extracted the other two molars, instead of attempting to treat what evidently he knew nothing about, I firmly believe the patient's life might have been prolonged.

